# The role of rumination in posttraumatic stress disorder and posttraumatic growth among adolescents after the wenchuan earthquake

**DOI:** 10.3389/fpsyg.2015.01335

**Published:** 2015-09-04

**Authors:** Xinchun Wu, Xiao Zhou, Yufei Wu, Yuanyuan An

**Affiliations:** ^1^School of Psychology, Institute of Developmental Psychology, Beijing Normal University, Beijing, China; ^2^Department of Mechanics and Engineering Science, Fudan University, Shanghai, China; ^3^School of Psychology, Nanjing Normal University, Nanjing, China

**Keywords:** posttraumatic stress disorder, posttraumatic growth, intrusive rumination, deliberate rumination, adolescent

## Abstract

Three hundred and seventy-six middle school students in Wenchuan County were assessed three and one-half years after the Wenchuan earthquake to examine the effects of rumination on posttraumatic stress disorder (PTSD) and posttraumatic growth (PTG). The results revealed that recent intrusive ruminations partly mediated the relationship between intrusive rumination soon after the earthquake with PTSD but not with PTG. Recent deliberate rumination partly mediated the relationship between intrusive rumination soon after the earthquake and PTG but not PTSD. Moreover, recent deliberate rumination also partly mediated the relationship between recent intrusive rumination with PTG but not with PTSD. Overall, intrusive rumination soon after the earthquake had an effect on PTSD but not on PTG through recent intrusive rumination and affected PTG but not PTSD through deliberate recent rumination. Furthermore, intrusive rumination soon after the earthquake affected PTG but not PTSD by recent deliberate rumination following recent intrusive rumination. More importantly, the present study also found that PTSD exhibited no relation to PTG. These results suggest that PTSD and PTG are influenced by different mechanisms, which further indicates that PTSD and PTG represent two separate dimensions of experience after adversity.

## Introduction

Traumatic events are notably common worldwide (Norris and Slone, 2007) and include sexual and physical assault, natural disasters, accidents, and combat. Posttraumatic stress disorder (PTSD) is the most common negative outcome after traumatic events (Campbell et al., 2007; Bal, 2008; Wang et al., 2012). Nevertheless, some positive outcomes, such as posttraumatic growth (PTG), have also been reported (Jin et al., 2014; Ying et al., 2014). Tedeschi and Calhoun (1995) suggested that PTG refers to positive life changes that follow traumatic events. More importantly, PTG and PTSD can co-exist in individuals following traumatic experiences (Tedeschi and Calhoun, 1996). Therefore, many studies have explored the relationship between PTSD and PTG. Some studies have shown that PTSD is negatively related to PTG (Davis et al., 1998; Frazier et al., 2001), whereas other studies have reported that PTSD is positively related to PTG (Park et al., 1996; Fontana and Rosenheck, 1998; Solomon and Dekel, 2007), and some researchers have suggested that PTSD has no relationship with PTG (Joseph et al., 1993; Cordova et al., 2001). These inconsistent results have attracted much attention from researchers and have been attributed to differences in designs and subjects of the relevant studies (Dekel et al., 2012). Few researchers have investigated the mechanisms that simultaneously affect PTSD and PTG to clarify the relationship between PTSD to PTG. The aim of this study was to examine and compare the possible mechanisms of the development of PTSD and PTG.

The shattered world assumption of Janoff-Bulman (2010) and the model of PTG of Calhoun and Tedeschi (2006) emphasize that posttraumatic cognitive activities occur in response to an initial traumatic event that challenges an individual’s beliefs about how the world works and their place in the world and that individuals then lose their framework for understanding. To rebuild the understanding of the posttraumatic world, individuals need to reexamine or repetitively think about the their beliefs before and after the trauma. This cognitive process is viewed as a process of “rumination” (Watkins, 2008; Cann et al., 2011) as has an important effect on posttraumatic reactions. However, previous studies have emphasized that some components of this ruminative process are risk factors for distress, whereas others can be crucial for growth (Janoff-Bulman, 1992; Linley and Joseph, 2004). Thus, Cann et al. (2010a) proposed a distinction between the two types of rumination or repetitive thinking about an event; i.e., intrusive rumination and deliberate rumination. The former represent thoughts that invade one’s cognitive world without the individual’s willingness and generally involve a negative focus on the trauma, whereas the latter involves deliberate reexamining of and contemplation about the event.

For most people, intrusive ruminations tend to occur soon after the traumatic event (Lindstrom et al., 2013). Soon after traumatic events, traumatic survivors can increase their attention to negative thoughts and feelings, which leads to negative expectations about the future and the activation of existing negative self-schemas (Scher et al., 2005) that cause the individuals to engage in intrusive rumination soon after events (Michl et al., 2013) and ultimately result in PTSD. Despite this process, in the view of the PTG model of Calhoun and Tedeschi (2006), intrusive rumination soon after a traumatic event can provide traumatic survivors with traumatic clues and opportunities for further cognitive processing of the traumatic event, which results in PTG. Thus, some researchers have suggested that intrusive rumination soon after a traumatic event is a risk factor for PTSD and also a predictive factor for PTG (Taku et al., 2008; Nightingale et al., 2010).

More importantly, engaging in intrusive rumination soon after a traumatic event can occur a long time after the traumatic event (Taku et al., 2008). In the assumption of a shattered world (Janoff-Bulman, 2010), intrusive rumination soon after a traumatic event aims to reduce the discrepancies between one’s current state and one’s desired state. If the rumination leads to a resolution of the discrepancy, then the rumination will stop. If the discrepancy cannot be resolved, the individual might continue to ruminate about it (Michl et al., 2013). However, if a discrepancy that was elicited by a traumatic event or the traumatic events cannot be reduced in a short amount of time, then an individual who experiences intrusive rumination soon after the event can continued to experience intrusive rumination, which can be viewed as recent intrusive rumination. Additionally, from the perspective of the PTG model of Calhoun and Tedeschi (2006), intrusive rumination soon after traumatic events can provide traumatic survivors with traumatic cues that provide opportunities for further cognitive processing of the traumatic events, which in turn result in recent deliberate rumination. Thus, it is likely that intrusive rumination soon after a traumatic event might predict recent intrusive and deliberate rumination.

Continued intrusive and deliberate thoughts play different roles in influencing the outcomes that follow a highly stressful experience (Calhoun and Tedeschi, 2006). Event-related intrusive rumination, which focuses on the negative effects of the traumatic event and negative affections, is more likely to be related to various types of posttraumatic distress, and event-related deliberate rumination, which is conducive to rebuilding the understandings of the world, the self and others after highly stressful events, is more likely to be related to eventual PTG (Affleck and Tennen, 1996; Taku et al., 2008; Cann et al., 2010b; Dunn et al., 2011). However, other studies have also found that continued intrusive rumination can expose people to traumatic cues and thereby encourage further cognitive processing of traumatic events, which results in PTG (Park and Fenster, 2004; Taku et al., 2009; Yanez et al., 2011), and continued deliberate rumination about traumatic events can change pathological thinking styles and reduce trauma-related fear, which in turn ameliorates PTSD symptoms (Ehlers and Steil, 1995; Paunovic and Öjst, 2001). Thus, continued intrusive rumination has been proposed to be a risk factor for PTSD and a predictive factor for PTG. Continued deliberate rumination has been proposed to be a protective factor against PTSD and a predictive factor for PTG. Additionally, it is assumed that the level of intrusive rumination is predictive of the level of deliberate rumination because intrusive rumination provides cues that can elicit deliberate rumination and can stimulate attempts to engage in more deliberate processing of the experience (Cann et al., 2010b).

While the theoretically expected co-existence of PTSD and PTG has been verified by empirical studies (Tedeschi and Calhoun, 1996), it remains unclear whether these two reactions are elicited by similar or different mechanisms. Furthermore, several theoretical predictions have been advanced to describe the effects of intrusive rumination soon after traumatic events and recent intrusive and deliberate ruminations on PTSD and PTG (Floyd, 2010; Lindstrom et al., 2013), but the predictive utilities of the theories that focus on the roles of recent intrusive and deliberate ruminations in the relationships of intrusive rumination soon after traumatic events with both PTSD and PTG have not been evaluated. Therefore, addressing this gap would advance (1) contemporary efforts that seek to clarify the relationship between PTSD and PTG (Solomon and Dekel, 2007) and (2) research about the roles of cognitive factors, particularly rumination, in the developmental processes of both PTSD and PTG (Chan et al., 2011).

The 2008 Wenchuan earthquake was the most devastating earthquake in China since 1976 and resulted in more than 69,000 deaths and 370,000 injuries, the majority of which involved adolescents. Compared to adults, adolescents are generally more vulnerable when they are exposed to trauma, and therefore, they often exhibit traumatic reactions following disasters (An et al., 2013). The present study examined how intrusive rumination soon after the Wenchuan earthquake and recent intrusive and deliberate rumination might be associated with both PTSD and PTG in the adolescent survivors of the Wenchuan earthquake. Based on the shattered world assumption (Janoff-Bulman, 2006) and the PTG model of Calhoun and Tedeschi (2006), we propose the following three hypotheses: (1) intrusive rumination soon after the earthquake would positively predict both PTSD and PTG, (2) recent intrusive and deliberate rumination would mediate the relationships of intrusive rumination soon after the earthquake with both PTSD and PTG, and (3) recent deliberate rumination would mediate the relationships of recent intrusive rumination with both PTSD and PTG.

## Materials and Methods

### Participants and Procedures

The present data were collected as part of a longitudinal study of psychological adjustment amongst the child survivors of the Wenchuan earthquake. For the present study, 376 adolescent survivors were randomly selected from several classes at the only two high schools in the county of Wenchuan in Nov, 2011. All the participants personally experienced this earthquake. On average, these participants were 15.98 years of age (SD = 1.64) with a range of 13–19 years old. One hundred ninety-eight of the participants (52.7%) were girls.

This study was approved by the Research Ethics Committee of Beijing Normal University and conducted according to the principals of the participating schools. In the selected classes, everyone who attended school on the date of the survey was recruited to participate. There were no exclusion criteria. Compensation was not provided. The purpose of the study and the voluntary nature of the students’ participation were highlighted before the survey, and written consent forms that emphasized the right of each participant to withdraw from the survey at any time were provided to participants and their parents. The researchers administered the questionnaire packets in a classroom setting without the presence of the teachers. The participants were initially asked to provide demographic information that included sex, age and ethnic group. They were asked to complete the remaining measures that assessed core belief challenge, rumination, PTG and PTSD. After the questionnaire packets were completed, the participants were told that school psychologists or teachers were available to provide any psychological/counseling services that they might need.

### Measures

#### Event-related Rumination Inventory

The modified Event-Related Rumination Inventory was used to measure intrusive and deliberate rumination (Zhou et al., 2014b). The items were rated on a 6-point scale that ranged from 0 (*not at all*) to 5 (*always*). The original Event-Related Rumination Inventory was developed by (Cann et al., 2011) and consists of 20 items. Some items from the original scale were reworded to form the modified ERRI, which has been testified to have a good reliability and validity in Chinese adolescent samples (Zhou et al., 2014a). The participants responded to each item two times, once based on their ruminations soon after the Wenchuan earthquake and once based on their recent ruminations. In the present study, the internal consistencies were good and were 0.90 for intrusive rumination soon after the earthquake and 0.88 and 0.89 for recent intrusive and deliberate rumination, respectively. The fit indices from a confirmatory factor analysis were good for rumination soon after the earthquake (*χ*^2^/df = 2.38, CFI = 0.92, TLI = 0.91, RMSEA = 0.061, SRMR = 0.049) and recent rumination (*χ*^2^/df = 2.57, CFI = 0.91, TLI = 0.90, RMSEA = 0.065, SRMR = 0.063).

#### Post-traumatic Growth Inventory

Posttraumatic growth was measured using a modified version of the Post-Traumatic Growth Inventory (Zhou et al., 2014a). The original Post-Traumatic Growth Inventory was developed by Tedeschi and Calhoun (1996) and consists of the following five subscales: personal strength, new possibilities, relating to others, appreciation of life, and spiritual change. Each of the 21 items is scored on a 6-point scale that ranges from 0 (*no change*) to 5 (*very great degree of change*). The PTGI has good internal consistency and good construct, convergent, and discriminate validities (Tedeschi and Calhoun, 1996). The revised PTGI includes the following three subscales for a total of 22 items: perceived changes in self, a changed sense of relationships with others, and a changed philosophy of life. The modified scale exhibits good reliability and construct validity for the sample of adolescent survivors of the Wenchuan earthquake. In this study, the internal reliability of the modified inventory was good (*α* = 0.90), and the fit indices from a confirmatory factor analysis were acceptable (*χ*^2^/df = 2.60, CFI = 0.90, TLI = 0.88, RMSEA = 0.065, SRMR = 0.049).

#### The Child PTSD Symptom Scale

Posttraumatic stress disorder symptom levels were assessed using the Child PTSD Symptom Scale (Foa et al., 2001) as applied to the Chinese population (Ying et al., 2014). This measure is a 17-item self-report scale that was designed to assess the occurrence and frequency of PTSD symptoms according to the Diagnostic and Statistical Manual of Mental Disorders in relation to the most distressing event experienced by an individual. In the present study, all of the items were translated into Chinese, and the respondents rated the frequencies of the symptoms during the previous 2 weeks on 4-point Likert scales that ranged from 0 (not at all/only once) to 3 (almost always or 5 or more times a week). The subscale scores range from 0 to 15 for intrusion symptoms, 0–21 for avoidance symptoms, and 0–15 for hyper-arousal symptoms. An overall severity score is generated by summing the scores for the three symptom types. In this sample, the scale demonstrated good internal consistency (*α* = 0.86), and the fit indices from a confirmatory factor analysis were good (*χ*^2^/df = 2.11, CFI = 0.92, TLI = 0.90, RMSEA = 0.054, SRMR = 0.054).

### Data Analysis Strategies

Descriptive analyses were conducted for all of the measures administered. Pearson correlations were calculated to examine the associations between the major variables.

The statistical analyses were conducted using Mplus 6.0 software (Muthén and Muthén, 2010). Because of participants’ omissions or data inputting problems, there are 0.71% missing data in the current sample. Missing data were handled with full-information maximum likelihood estimates (FIML) in structural models. Compared to conventional methods of dealing with missing data (e.g., listwise deletion and pairwise deletion), the results produced by the FIML method are less biased and more reliable (Muthén et al., 1987). Thus, the present structural equation models (SEM) analysis was based on the total sample of 376 individuals.

Additionally, to evaluate the model fit, we used chi-square values, the comparative fit index (CFI), the Tucker–Lewis index (TLI), the root mean square error of approximation (RMSEA), and the standardized root mean residual (SRMR). A non-significant chi-square indicates good model-data fit. The general cutoffs for accepting a model are equal to or greater than 0.90 for the CFI and TLI and equal to or less than 0.08 for the RMSEA and SRMR (Wen et al., 2004b).

**FIGURE 1:**
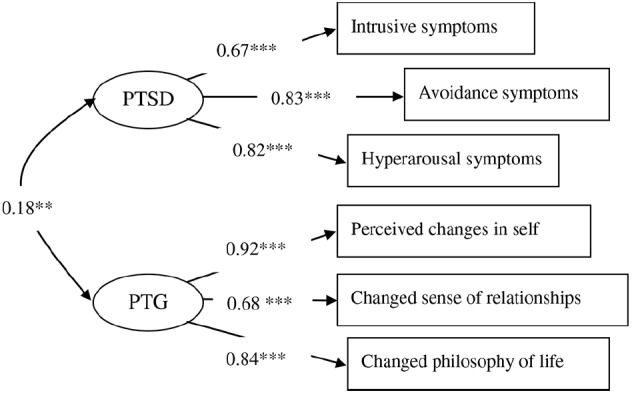
**The measurement model.** **p < 0.01, ***p < 0.001.

We built SEM to examine the effects of intrusive rumination soon after the earthquake, recent intrusive rumination, recent deliberate rumination on PTSD and PTG. SEM models have two basic elements: a measurement component (“measurement model”), and a structural component (“structural model”). Although both the measurement and structural components can be evaluated simultaneously in SEM (Anderson and Gerbing, 1988) strongly recommended that the measurement model fit be evaluated prior to proceeding to an evaluation of the full model (i.e., the measurement and structural models together). This stepwise procedure offers the safeguard of explicitly verifying the acceptability of the measurements of the constructs before proceeding to the evaluation of relationships between the constructs. If the measurement model fails to fit the observed data, then the operationalization of the constructs into the observed measures has not been successful. There is little sense in proceeding to evaluating the relationships among constructs (i.e., the structural model) if the measurement model is inadequate (Hays et al., 1994). Thus, we first assessed the measurement model (see Figure 1) and then assessed the structural model.

**FIGURE 2:**
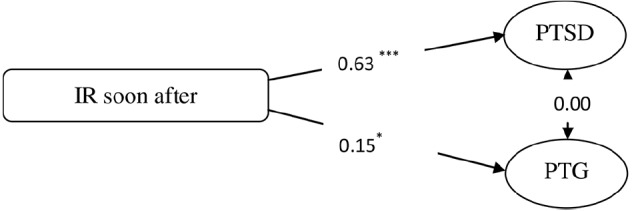
**The direct effect model (Model 1).** *p < 0.05, ***p < 0.001.

**FIGURE 3:**
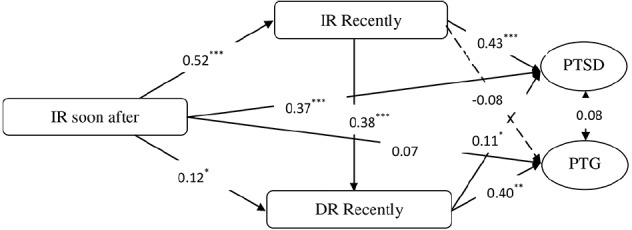
**The multiple indirect effects model (Model 2).** *p < 0.05, **p < 0.01, ***p < 0.001.

Next, according to mediating effects test procedures (Wen et al., 2004a), we applied an SEM approach to assess the following two SEM models: (1) a direct effect model (M1, see Figure 2) with structural paths from intrusive rumination soon after the earthquake to PTSD and PTG such that PTSD and PTG were assumed to be related because of their observed co-existence in trauma survivors (Tedeschi and Calhoun, 1996), and (2) a model based on M1 and the model of PTG proposed by (Calhoun and Tedeschi, 2006) in which we inserted mediators (e.g., recent intrusive and deliberate rumination) between intrusive rumination soon after the earthquake and PTSD/PTG and added one path from recent intrusive rumination to recent deliberate rumination to establish a multiple indirect effects model (M2, see Figure 3). Moreover, to test the significance of this indirect effect in M2, we conducted bias-corrected bootstrap tests with a 95% confidence interval (Gootzeit and Markon, 2011).

## Results

### Descriptive Statistics and Correlations Between Measures

To describe the study sample, the means and standard deviations of the measures are presented in Table 1. The correlations between all of the main variables are also presented in Table 1. The Correlations between these measures were positive and significant and ranged from 0.16 to 0.57.

**TABLE 1:** **Means, standard deviations and correlations between immediately intrusive rumination soon after the events, recent intrusive rumination, recent deliberate rumination, PTSD and PTG**.

****	**M ± SD**	**1**	**2**	**3**	**4**	**5**
1. IR soon after	10.25 ± 5.93	1.00				
2. IR recently	6.33 ± 4.82	0.52**	1.00			
3. DR recently	9.34 ± 6.04	0.32**	0.44**	1.00		
4. PTSD	13.03 ± 7.23	0.52**	0.57**	0.39**	1.00	
5. PTG	61.09 ± 18.98	0.16**	0.14**	0.35**	0.17**	1.00

** indicates the correlation was significant at the 0.01 level (2-tailed). IR soon after, intrusive rumination soon after events; IR recently, recent intrusive rumination; DR recently, recent deliberate rumination.

### Structural Equation Model Analyses

#### Phase 1: Measurement Model Results

We built a measurement model that included the two latent variable constructs of PTSD and PTG. Next, the PTSD latent variable was evaluated according to the scores for the CPSS subscales of Intrusion, Avoidance and Hyper-arousal (Foa et al., 2001), whereas the PTG latent variable was evaluated in terms of perceived changes in the self, a changed sense of relationships with others, and a changed philosophy of life (Zhou et al., 2014a,b). In this measurement model (Figure 1), correlations were specified between PTSD and PTG. The factor loadings of the manifest indicators on their respective latent variables were estimated freely. The model fit the data well [*χ*2(8) = 16.001, CFI = 0.992, TLI = 0.984, RMSEA (90% CI) = 0.052(0.009–0.088), SRMR = 0.032]. The results indicated that the measurement model was good and applicable to the analysis of the SEM.

#### Phase 2: Structural Model Results

In this phase, we formulated two structural models using the measurement model mentioned above. First, we built a direct effects model (M1), which demonstrated that intrusive rumination soon after the event had direct effects on PTSD and PTG. The model fit the data well [*χ*^2^(11) = 19.34, *CFI* = 0.992, *TLI* = 0.985, RMSEA (90% CI) = 0.045 (0.000–0.077), *SRMR* = 0.027]. The path analysis revealed that there were significant direct effects of the intrusive rumination soon after the earthquake on PTSD and PTG, and the relation between PTSD and PTG was marginally significant (*p* = 0.051).

Second, based on the direct effects model, we added recent intrusive and deliberate rumination to the relationships of intrusive rumination soon after the earthquake with both PTSD and PTG. Moreover, based on the PTG model of (Calhoun and Tedeschi, 2006) and the *Pearson* correlations observed in the present data, we added a path from recent intrusive rumination to recent deliberate rumination and established a multiple indirect effects model (M2, see Figure 3). This model exhibited good fit, *χ*^2^(17) = 36.07, *CFI* = 0.989, *TLI* = 0.976, RMSEA (90% CI) = 0.056 (0.030–0.082), *SRMR* = 0.029. These results indicated that the M2 was acceptable.

Next, to evaluate the significance levels of the indirect effects in M2, we conducted bias-corrected bootstrap tests with a 95% confidence interval to determine the significance of the indirect effects (Gootzeit and Markon, 2011). First, 5000 bootstrap samples were created from the original data set (*n* = 376) using random samples with replacement. Model 2 was conducted 5000 times with these samples to yield 5000 estimations of each indirect path coefficient. If the 95% confidence interval for the estimate of indirect path coefficient does not include 0, it could be concluded that the indirect path coefficient is significant.

Table 2 illustrates the results of bias-corrected bootstrap tests, and these results revealed that intrusive rumination soon after the earthquake had an effect on PTSD but not on PTG through recent intrusive rumination, which indicates that recent intrusive rumination partly mediated the relationship of intrusive rumination soon after the earthquake with PTSD but not the relationship between intrusive rumination soon after the earthquake and PTG. Intrusive rumination soon after the earthquake affected PTG but not PTSD through recent deliberate rumination, which indicates that recent deliberate rumination partly mediated the relationship between intrusive rumination soon after the earthquake and PTG but not the relationship between intrusive rumination soon after the earthquake and PTSD. Additionally, there were multiple mediating effects by which intrusive rumination soon after the earthquake affected PTG but not PTSD by recent deliberate rumination following recent intrusive rumination. More importantly, the present study also found that PTSD exhibited no relation to PTG.

**TABLE 2:** **Bias-corrected bootstrap tests of the mediating effects**.

		**95% CI**	
**Paths**	**β**	**Low**	**High**
Direct paths			
IR soon after-PTSD	0.37***	0.28	0.47
IR soon after-PTG	0.07	–0.04	0.19
Indirect paths			
IR soon after—IR recently-PTSD	0.23***	0.17	0.28
IR soon after–IR recently-PTG	–0.04	–0.10	0.02
IR soon after—DR recently-PTSD	0.01	–0.002	0.03
IR soon after—DR recently-PTG	0.05*	0.01	0.09
IR soon after—IR recently—DR recently-PTSD	0.02	0.003	0.04
IR soon after—IR recently—DR recently-PTG	0.08***	0.05	0.11

IR soon after, intrusive rumination soon after the events; IR recently, recent intrusive rumination; DR recently, recent deliberate rumination. *p < 0.05, ***p < 0.001.

## Discussion

In this study, we constructed a multiple indirect effects model to examine the relationships between intrusive rumination soon after traumatic events, recent intrusive rumination, recent deliberate rumination, PTSD and PTG in a sample of adolescent survivors of the Wenchuan earthquake. The adolescents’ intrusive ruminations soon after the earthquake positively predicted PTSD; i.e., the adolescents who experienced more intrusive rumination soon after the earthquake exhibited more severe PTSD symptoms, which is consistent with the results of Nightingale et al.’s (2010) study. More importantly, intrusive rumination soon after the earthquake also positively predicted PTSD via recent intrusive rumination. Engaging in intrusive rumination soon after the earthquake can increase individuals’ negative thoughts about the causes and/or consequences of those events or distressing/negative affect (Watkins, 2008), reduce the capacity to disengage attention from negative emotional information (Joormann, 2006), and result in the difficulties in generating good solutions to problems (Ward et al., 2003; Watkins and Moulds, 2005). Thus, adolescents who report more PTSD symptoms are also more prone to pessimistic thinking can increase the opportunities for prolonged intrusive rumination (Nolen-Hoeksema et al., 1994), which in turn maintains and increases the severity of PTSD symptoms. These results also suggest that the adolescents’ cognitive discrepancies were not resolved by intrusive rumination soon after the earthquake.

Inconsistent with previous studies (Taku et al., 2008; Nightingale et al., 2010), the present study found that intrusive rumination soon after the earthquake had a non-significant effect on PTG. Moreover, intrusive rumination soon after the earthquake had a non-significance effect on PTG via recent intrusive rumination. An possible explanation for these findings is that intrusive rumination did not distract the individuals from the negative outcomes and effects of the traumatic events (Nolen-Hoeksema and Morrow, 1993); i.e., the adolescents who engaged in intrusive rumination focused on the negative aspects of the earthquake and found it difficult to think positively about the earthquake; thus intrusive rumination had a non-significant direct effect on PTG regardless of whether it occurred soon after the earthquake or recently. Despite this finding, intrusive rumination both soon after the earthquake and recently provided clues that the adolescents thought positively about and elicited subsequent deliberate rumination that, in turn, was conducive to the reconstruction of the understanding of the posttraumatic world and the benefits of that process and eventually increased the opportunity for PTG. These results are consistent with the model of PTG from Calhoun and Tedeschi (2006).

Other interesting findings of this study were that recent deliberate rumination have a positive and significant effect on PTSD, which be inconsistent with previous studies (Ehlers and Steil, 1995; Paunovic and Öjst, 2001). Here, when adolescents engage into recent deliberate rumination, they may actively expose themselves to the traumatic clues and try to reconstruct their cognition about life and the world. However, being exposed to traumatic experiences may also elicit individuals’ negative psychological reactions, and in turn result in PTSD.

In SEM, we found that intrusive rumination soon after the earthquake had a positive and significant effect on PTSD via recent deliberate rumination. We further used bias-corrected bootstrap test with a 95% confidence interval to determine whether the indirect effect was truly significant. However, the result showed that this indirect path was non-significant, indicating that intrusive rumination soon after the earthquake could not predict PTSD via recent deliberate rumination. Here, the traumatized adolescents who engaged in deliberate rumination indicated that they had been continuing to struggle with and to understand the trauma (Taku et al., 2008). During this process, adolescents are typically not cognitively overwhelmed by trauma-related cues and might not present as symptomatic for PTSD.

Soon after traumatic events, trauma survivors always engage in intrusive rumination, which has important effects on subsequent cognitive activities, particularly recent intrusive and deliberate rumination that, in turn predict PTSD or PTG. The present study found that intrusive rumination soon after the earthquake led to both PTSD and PTG, and recent intrusive rumination played a role in the former, while recent deliberate rumination played a role in the latter. These findings further support the assumption of (Chan et al., 2011) that the pathways to PTSD and PTG are different. From this perspective, this study demonstrated that there are different predictive factors that have initial effects on both PTSD and PTG and that these two outcomes later diverge and proceed to be manifested via different developmental processes. These results further indicate that PTSD and PTG are separate, independent dimensions of experience that follow adversity (Linley and Joseph, 2004).

Although most of this study’s theoretically derived predictions were supported, several major design and measurement limitations must be acknowledged. One major limitation is the cross-sectional design, which means that the findings do not indicate causality or a temporal sequence. A second limitation is that the reports by the participants about their ruminations “soon after the event” and “recently” might have been biased by the inability to accurately recall the thought processes that occurred in the past (Taku et al., 2008). In addition, all variables were measured by self-report scales, thus associations between the main measures might be affected by common-method variance. Moreover, the construct of PTG measured by the current modified version of the Post-Traumatic Growth Inventory is different from that measured by the original one (three dimensions vs. five dimensions), so comparisons between the current and previous findings must be made with caution.

Despite these limitations, to our knowledge, this study is among the first to examine the cognitive processing differences between PTSD and PTG. Furthermore, this study contributes new knowledge to previous theoretical and empirical studies of the relationship between PTSD and PTG that have indicated that PTSD and PTG can independently co-exist in traumatized adolescents. From the intervention and health-enhancement perspectives, this study also highlights important implications for adolescent survivors of the Wenchuan earthquake. Substantial growth can still occur even in the face of distressing symptoms, and treating the latter should not impede the former. Clinical efforts should focus on the enhancement of recent deliberate rumination even as intrusive rumination soon after the earthquake and recently are decreased.

### Conflict of Interest Statement

The authors declare that the research was conducted in the absence of any commercial or financial relationships that could be construed as a potential conflict of interest.
